# Letter to Editor: Clinical performance of a machine learning-based model for detecting lymph node metastasis in papillary thyroid carcinoma: a multicenter study

**DOI:** 10.1097/JS9.0000000000002949

**Published:** 2025-07-18

**Authors:** Dingliu He, Shiliang Ji

**Affiliations:** a Department of Clinical Nutrition, Yancheng Clinical College of Xuzhou Medical University, The First People’s Hospital of Yancheng, Yancheng, China; b Suzhou Research Center of Medical School, Suzhou Hospital, Affiliated Hospital of Medical School, Nanjing University, Suzhou, China

*Dear Editor,*

We have carefully reviewed the recently published article in your journal entitled *“Clinical Performance of a Machine Learning-Based Model for Detecting Lymph Node Metastasis in Papillary Thyroid Carcinoma: A Multicenter Study”*^[1]^. The study presents a predictive model with promising potential for preoperative risk assessment. While we acknowledge the authors’ efforts and recognize the value of their contribution, we would like to raise several critical points regarding the study’s design, interpretability, and clinical applicability for the authors’ and readers’ consideration.

## Limitations in sample size and external validation

The authors selected four candidate genes by identifying overlapping differentially expressed genes from the TCGA cohort and their dataset. However, the two cohorts represent different populations; for example, TCGA includes a significant proportion of White individuals, whereas the genetic landscape of thyroid cancer varies considerably across ethnic groups^[2]^. For studies targeting Asian populations, it is more appropriate to ensure that the training set consists of ethnically matched cohorts. Moreover, the external validation cohorts (Nantong, Zhongshan, and Xuzhou) utilized different sequencing platforms (microarray vs. RNA-seq), which can introduce batch effects. Although normalization was performed, the authors did not describe in detail how batch effects were mitigated or how correction effectiveness was assessed. We suggest including additional clarification and, where applicable, applying established methods such as ComBat along with relevant validation metrics.

## Biological plausibility of selected features

The final model is based on three genes: *RPS4Y1, PKHD1L1*, and *CRABP1*. Although *CRABP1* and *PKHD1L1* have been associated with PTC and metastasis in prior studies^[3,4]^, the model’s performance hinges on these three genes collectively. Understanding the biological interactions among them in the context of thyroid cancer is crucial for interpreting the high predictive accuracy observed. We recommend that future work explore the functional relevance of these genes in tumor progression and metastasis, to reinforce the model’s biological rationale beyond statistical associations.

## Model interpretability and clinical feasibility

The use of SHAP values helps improve the transparency of the model by elucidating gene-level contributions to predictions. However, SHAP only reflects statistical correlation, not causation. Furthermore, the model lacks a clearly defined probability threshold and corresponding clinical decision-making criteria, limiting its practical utility. In real-world settings, predictive probabilities alone are insufficient for surgical planning. The authors are encouraged to elaborate on how the model would be integrated into preoperative workflows, including decision thresholds and risk communication strategies.

## Comparative advantage over conventional methods requires quantification

Although the study suggests that the model outperforms traditional ultrasound, it does not provide a systematic comparison with ultrasound results. A quantitative analysis against the clinical gold standard is lacking. To better demonstrate the model’s clinical utility, we recommend the inclusion of comparative metrics such as ROC curve analysis and the Net Reclassification Index to evaluate the extent of improvement in diagnostic accuracy. Additionally, it is important to note that the gene expression data used in the model were obtained from postoperative specimens. Since lymph node dissection is performed intraoperatively, gene sequencing data cannot inform real-time surgical decisions. The authors should address this temporal limitation explicitly.

In summary, this study offers a valuable exploration into preoperative risk stratification for thyroid cancer using machine learning. However, for meaningful clinical translation, further improvements are needed in terms of cohort design, mechanistic validation, model transparency, and real-world applicability. We appreciate the innovative direction proposed by the authors and look forward to seeing continued optimization in future research to support the integration of AI-assisted tools in thyroid cancer management.

## Data Availability

Not applicable.

## Research registration unique identifying number (UIN)

There are no conflicts of interest.

## References

[1] Liu W Zheng J Han L. Clinical performance of a machine learning-based model for detecting lymph node metastasis in papillary thyroid carcinoma: a multicenter study. Int J Surg 2025;111:4062–67. 40265473 10.1097/JS9.0000000000002400 PMC12165525

[2] Zhou SL Guo YP Zhang L. Predicting factors of central lymph node metastasis and BRAF(V600E) mutation in Chinese population with papillary thyroid carcinoma. World J Surg Oncol 2021;19:211. 34256769 10.1186/s12957-021-02326-y PMC8278623

[3] Zheng C Quan R Xia EJ. Original tumour suppressor gene polycystic kidney and hepatic disease 1-like 1 is associated with thyroid cancer cell progression. Oncol Lett 2019;18:3227–35. 31452800 10.3892/ol.2019.10632 PMC6676403

[4] Wang QX Chen ED Cai YF. A panel of four genes accurately differentiates benign from malignant thyroid nodules. J Exp Clin Cancer Res 2016;35:169. 27793213 10.1186/s13046-016-0447-3 PMC5084448

[5] Agha R Mathew G Rashid R. Transparency In The reporting of Artificial Intelligence – the TITAN guideline. Prem J Sci 2025;10:100082.

